# Gene therapy for human glioblastoma using neurotropic JC virus-like particles as a gene delivery vector

**DOI:** 10.1038/s41598-018-19825-w

**Published:** 2018-02-02

**Authors:** Chun-Nun Chao, Yu-Hsuan Yang, Mu-Sheng Wu, Ming-Chieh Chou, Chiung-Yao Fang, Mien-Chun Lin, Chien-Kuo Tai, Cheng-Huang Shen, Pei-Lain Chen, Deching Chang, Meilin Wang

**Affiliations:** 1 Department of Pediatrics, Ditmanson Medical Foundation Chiayi Christian Hospital, Chiayi, Taiwan; 2 Institute of Molecular Biology, National Chung Cheng University, Chiayi, Taiwan; 3 Department of Medical Research, Ditmanson Medical Foundation Chiayi Christian Hospital, Chiayi, Taiwan; 4 Department of Urology, Ditmanson Medical Foundation Chiayi Christian Hospital, Chiayi, Taiwan; 5 Department of Medical Laboratory Science and Biotechnology, Central Taiwan University of Science and Technology, Taichung, Taiwan; 6 Department of Microbiology and Immunology, School of Medicine, Chung-Shan Medical University and Clinical Laboratory, Chung-Shan Medical University Hospital, Taichung, Taiwan

## Abstract

Glioblastoma multiforme (GBM), the most common malignant brain tumor, has a short period of survival even with recent multimodality treatment. The neurotropic JC polyomavirus (JCPyV) infects glial cells and oligodendrocytes and causes fatal progressive multifocal leukoencephalopathy in patients with AIDS. In this study, a possible gene therapy strategy for GBM using JCPyV virus-like particles (VLPs) as a gene delivery vector was investigated. We found that JCPyV VLPs were able to deliver the GFP reporter gene into tumor cells (U87-MG) for expression. In an orthotopic xenograft model, nude mice implanted with U87 cells expressing the near-infrared fluorescent protein and then treated by intratumoral injection of JCPyV VLPs carrying the thymidine kinase suicide gene, combined with ganciclovir administration, exhibited significantly prolonged survival and less tumor fluorescence during the experiment compared with controls. Furthermore, JCPyV VLPs were able to protect and deliver a suicide gene to distal subcutaneously implanted U87 cells in nude mice via blood circulation and inhibit tumor growth. These findings show that metastatic brain tumors can be targeted by JCPyV VLPs carrying a therapeutic gene, thus demonstrating the potential of JCPyV VLPs to serve as a gene therapy vector for the far highly treatment-refractory GBM.

## Introduction

Glioblastoma multiforme (GBM) is the most common and lethal type of malignant primary brain tumor, representing 20% of all intracranial tumors. Unlike breast cancer, prostate cancer, and colorectal cancer, whose survival rates have significantly increased over the past decades, survival rates for highly malignant gliomas have remained stubbornly low^1^. In 2005, the standard-of-care treatment for GBM was changed to surgical resection followed by adjuvant radiotherapy with concomitant temozolomide chemotherapy. This treatment improved median survival from 12.1 months for surgery plus radiotherapy alone to 14.6 months^2,3^. Since then, no major progress has been made in improving the effectiveness of GBM treatment, with only 2% of patients surviving longer than three years^4^. Almost all patients experience tumor recurrence several months after treatment and develop resistance to temozolomide. Therefore, besides standard therapy, it is imperative that new, effective treatment methods need to be developed, such as gene therapy.

JC virus (JCPyV) infects glial cells and oligodendrocytes in the central nervous system and causes fatal progressive multifocal leukoencephalopathy (PML) in AIDS patients^5,6^. The capsid of JCPyV is made up of three proteins, VP1, VP2, and VP3, of which the major capsid protein VP1 forms the outermost layer of the virus and is responsible for receptor binding^7^. The discovery in 1970 that the coat protein of polyomavirus can transfer host genes to another animal cells^8^ launched research into using this protein in gene therapy applications. More recently, we found that simultaneously transforming a JCPyV VP1 expression plasmid^9^ and another expression plasmid into *E. coli* resulted in the assembly of virus-like particles (VLPs) in which the second expression plasmid DNA was packaged^10^. This *in vivo* DNA packaging method not only enables the mass production of VLPs but also greatly increases the efficiency of gene transfer to cells by the VLPs^10–13^. VLPs composed of JCPyV VP1 are similar to viruses in structure, hemagglutination activity, and ability to infect cells and enter the cell nucleus^14–16^. Previous studies in experimental animals showed that JCPyV can induce several types of brain tumors, such as oligoastrocytomas, glioblastomas, and medulloblastomas^17,18^. The JCPyV early DNA sequence was detected in malignant glioma and glioblastoma cells from patients, and expression of the viral early protein T-antigen was observed in the nuclei of a proportion of brain tumors^19,20^. These findings suggest that human glioblastoma cells are susceptible to infection by JCPyV, and that it may be feasible to use JCPyV VLPs to deliver therapeutic genes for treating human GBM.

Recent advances in fluorescent protein research have made it possible to label tumor cells with fluorescent markers and track tumor growth, metastasis, and angiogenesis in small animals^21,22^. Tumor cells marked by near-infrared fluorescent protein (iRFP) can be grown in tissue culture or in mice and be tracked accurately for cell proliferation *in vitro* and tumor growth *in vivo*^23^. Since the laser excitation spectrum of fluorescence molecular tomography (FMT) already encompasses that of iRFP, we employed intravital FMT imaging to continually and directly monitor the growth of intracranially inoculated iRFP-expressing human glioblastoma (U87-L-iRFP) cells in nude mice. In this orthotopic animal model of malignant glioma, quantification of iRFP was used to track tumor growth and tumor response to gene therapy. Survival analysis was also performed to examine the effect of gene therapy. In addition, we have also tested whether JCPyV VLPs given by tail vein injection could protect and deliver their packaged suicide gene to subcutaneous human glioblastoma xenografts in nude mice and inhibit tumor growth.

## Results

### Gene delivery into U87-MG cells for expression by JCPyV VLPs

**Figure 1:**
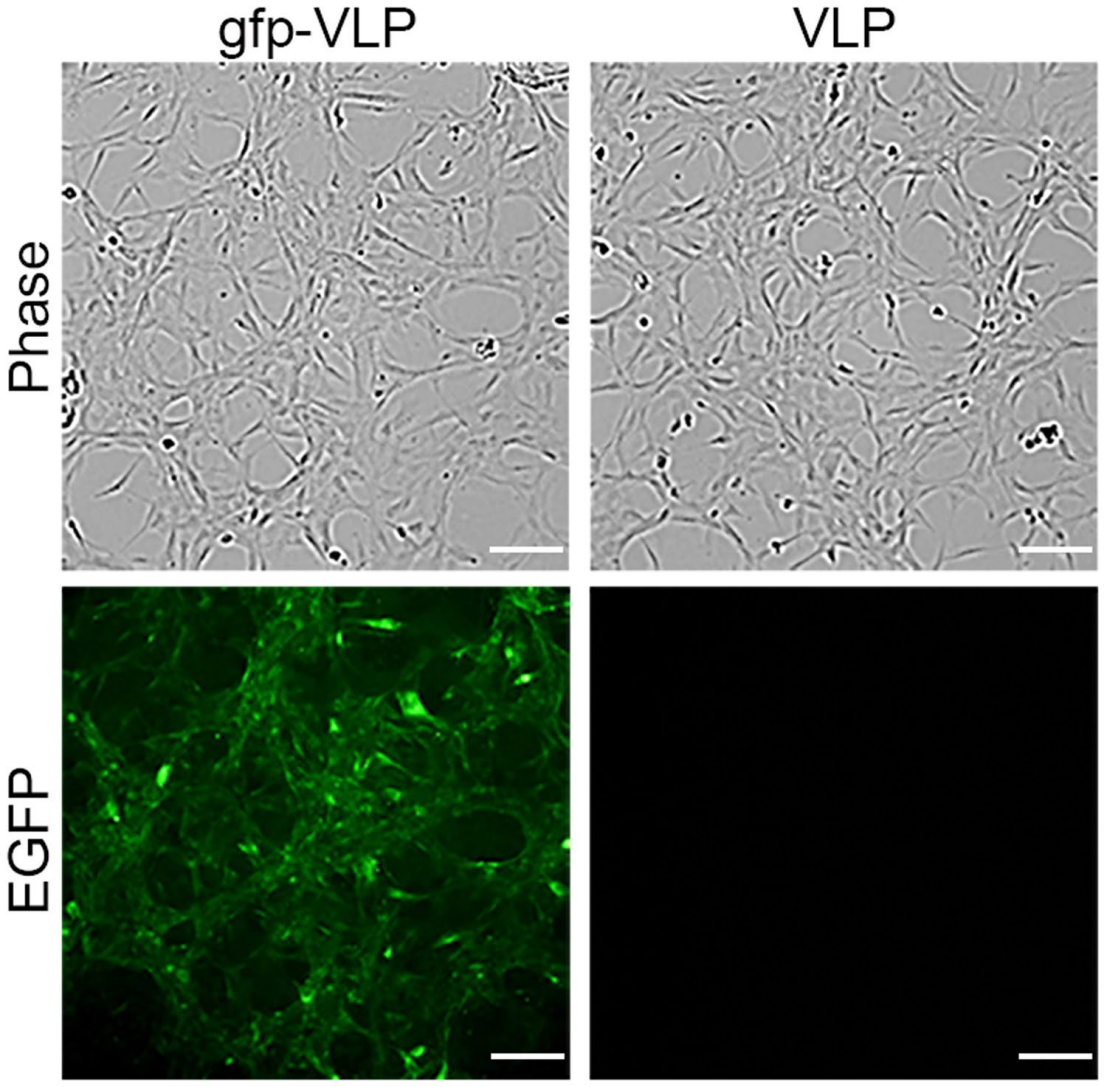
Transduction of the EGFP gene into human glioblastoma U87 cells by JCPyV VLPs. U87-MG cells were transduced with control VLPs or with gfp-VLPs. The expression of green fluorescent protein in the transduced cells was visualized with a fluorescence microscope at 72 h post-transduction. Scale bar = 100 μm.

To determine whether JCPyV VLPs could deliver genes into human glioblastoma cells for expression, U87-MG cells were transduced with JCPyV VLPs carrying the green fluorescent protein (GFP) gene (gfp-VLPs) and examined 72 h later for GFP expression under a fluorescence microscope. Compared with cells transduced with control (empty) VLPs, gfp-VLP transduced U87-MG cells clearly exhibited green fluorescence (Fig. 1), indicating that JCPyV VLPs were able to deliver the GFP reporter gene into human glioblastoma cells for expression.

### Cytotoxicity of U87-MG cells induced by tk-VLPs transduction and GCV treatment

**Figure 2:**
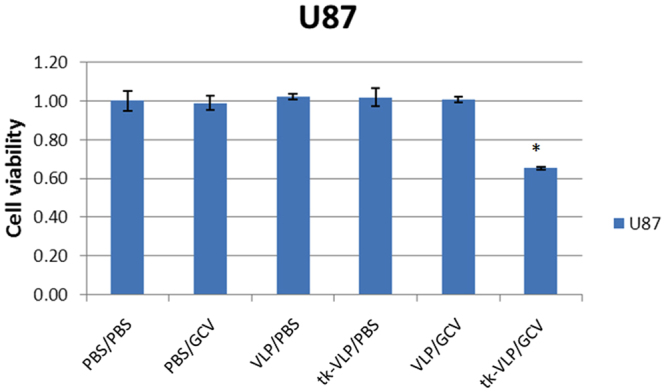
Assessment of cytotoxicity of tk-VLPs combined with GCV on U87 cells post-transduction. U87-MG cells were treated with PBS/PBS, PBS/GCV, control VLP/PBS, tk-VLP/PBS, control VLP/GCV, or the tk-VLP/GCV combination, and the viability of the cells was determined by the CCK-8 assay 72 h after treatment. Bar diagrams show the mean ± SD obtained from triplicate experiments. *p < 0.05.

In order to test whether JCPyV VLPs could deliver a packaged herpes simplex virus thymidine kinase suicide gene (tk-VLPs) to cultured U87-MG cells and achieve cytotoxicity in combination with ganciclovir (GCV) treatment before conducting *in vivo* study, U87-MG cells were transduced with tk-VLPs and treated with GCV, and the Cell Counting Kit-8 (CCK-8) assay was performed 72 h later to assess cell viability. The results show that relative to phosphate-buffered saline (PBS), VLPs, or GCV only treatment and control VLP/GCV combination, tk-VLPs plus GCV reduced the viability of the cells significantly (Fig. 2), indicating that JCPyV VLPs delivered thymidine kinase suicide gene into human glioblastoma cells and induced cytotoxicity in combination with GCV. A lower tk-VLP/GCV induced cell killing *in vitro* (about 40%) may be due to the doubling time of cell proliferation which is about 24 hours and the cells were harvest at 72 hours post-transduction. Therefore, only the parental cells were transduced by tk-VLPs but the newly proliferated cells were not.

### Examination of mice injected with U87 cells pre-mixed with tk-VLPs

**Figure 3:**
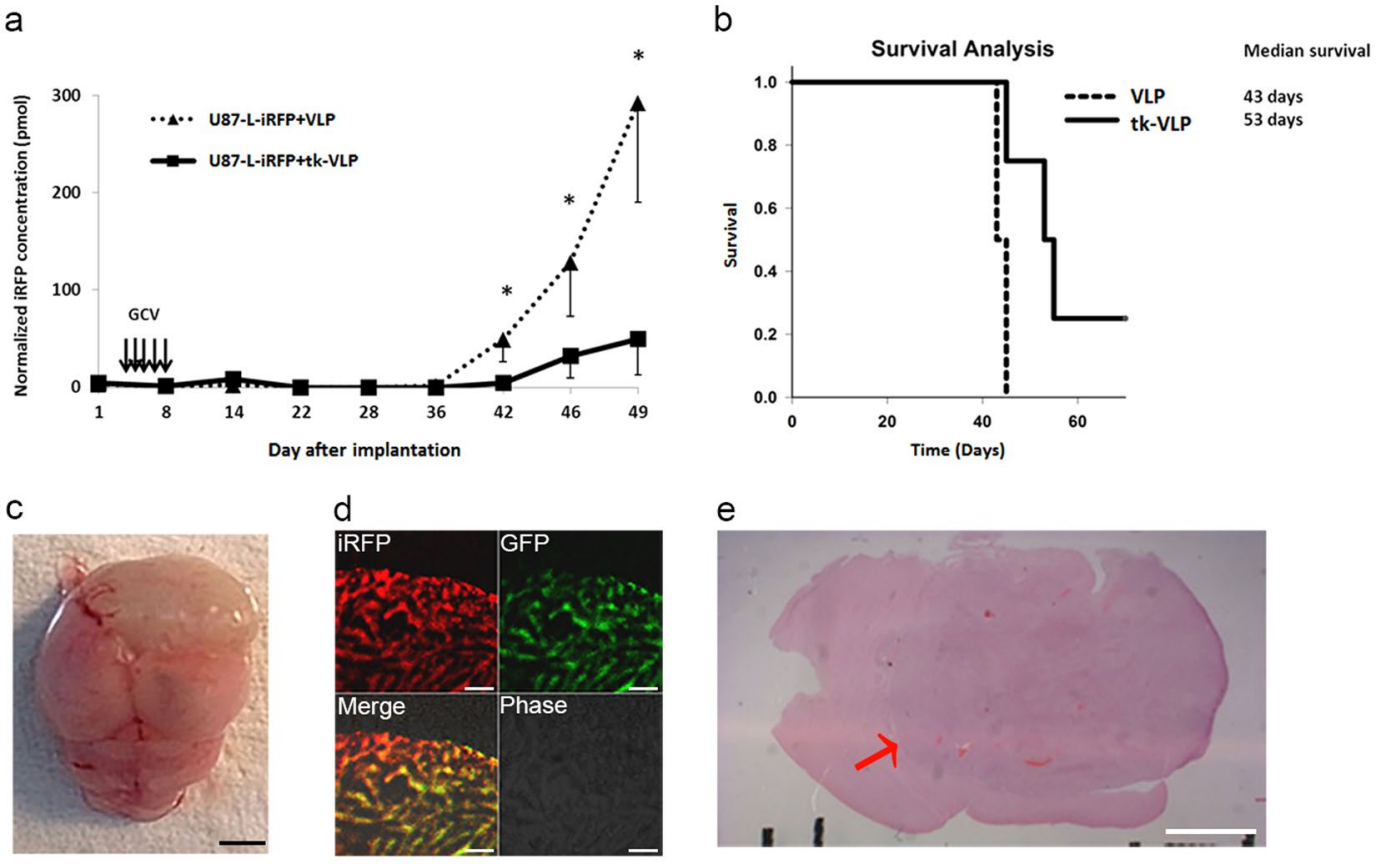
Analysis of mice injected with U87 cells pre-mixed with tk-VLPs in an orthotopic gioma model. (**a**) Tumor iRFP fluorescence over time. U87-L-iRFP cells were mixed with control VLPs or tk-VLPs and then injected into the right brain of nude mice in combination with GCV treatment. At different time points, the two groups of mice were subjected to *in vivo* FMT imaging. Data were means ± SD. n = 4, Mann–Whitney U test, *p* = 0.021 * (day 42), 0.021 * (day 46), 0.021 * (day 49). (**b**) Kaplan–Meier survival analysis of mice with orthotopic glioma treated with control VLPs or tk-VLPs. Log-rank test, *p* = 0.028. Analysis of tissue from a representative mouse in the treated group (cells pre-mixed with tk-VLPs) at the end of experiment including the gross appearance of a tumor-bearing brain (**c**), scale bar = 25 mm; the confocal microscopic images of double fluorescence of the tumor (**d**), scale bar = 60 μm; and H&E-stained brain section (**e**) from around the needle insertion point, with the location of the tumor indicated by a red arrow. Scale bar = 2 mm.

In order to use near-infrared fluorescence to track the growth of intracranial tumors, this fluorescence must be stably expressed by human glioblastoma cells, U87-L-iRFP cells in this case. A confocal microscope with laser excitation at 488 nm and 633 nm was used to observe the fluorescence of GFP and iRFP, respectively, in U87-L-iRFP cells. In contrast to the non-fluorescent parental U87-MG cells, all of the U87-L-iRFP cells observed were doubly fluorescent (Supplementary Fig. 1). U87-L-iRFP cells were then used to establish an orthotopic animal model of glioma for testing tk-VLPs as a gene therapy strategy. To ensure that the VLPs could fully interact with the tumor cells, U87-L-iRFP cells were mixed with tk-VLPs before being injected into the right brain of nude mice, followed by supplementation with GCV. Compared with mice injected with tumor cells pre-mixed with control VLPs, tk-VLP mixture–injected mice had significantly less intracranial tumor fluorescence from day 42 on as determined through *in vivo* FMT imaging (Fig. 3a and Supplementary Fig. 2) and had significantly better survival (Fig. 3b). The mean weight of mice before the onset of disease did not differ between the control group and the experimental group (Supplementary Table 1). When brains removed from nude mice that were euthanized after disease onset were sectioned and examined under a confocal microscope, iRFP and GFP double fluorescence were observed where tumors were located (Fig. 3d and e). Thus, these results show that U87-L-iRFP cells stably expressed iRFP in an *in vivo* setting, and provide histopathological evidence supporting the reliability of FMT tumor imaging.

### Examination of mice with orthotopic glioma treated by intratumoral injection of tk-VLPs

**Figure 4:**
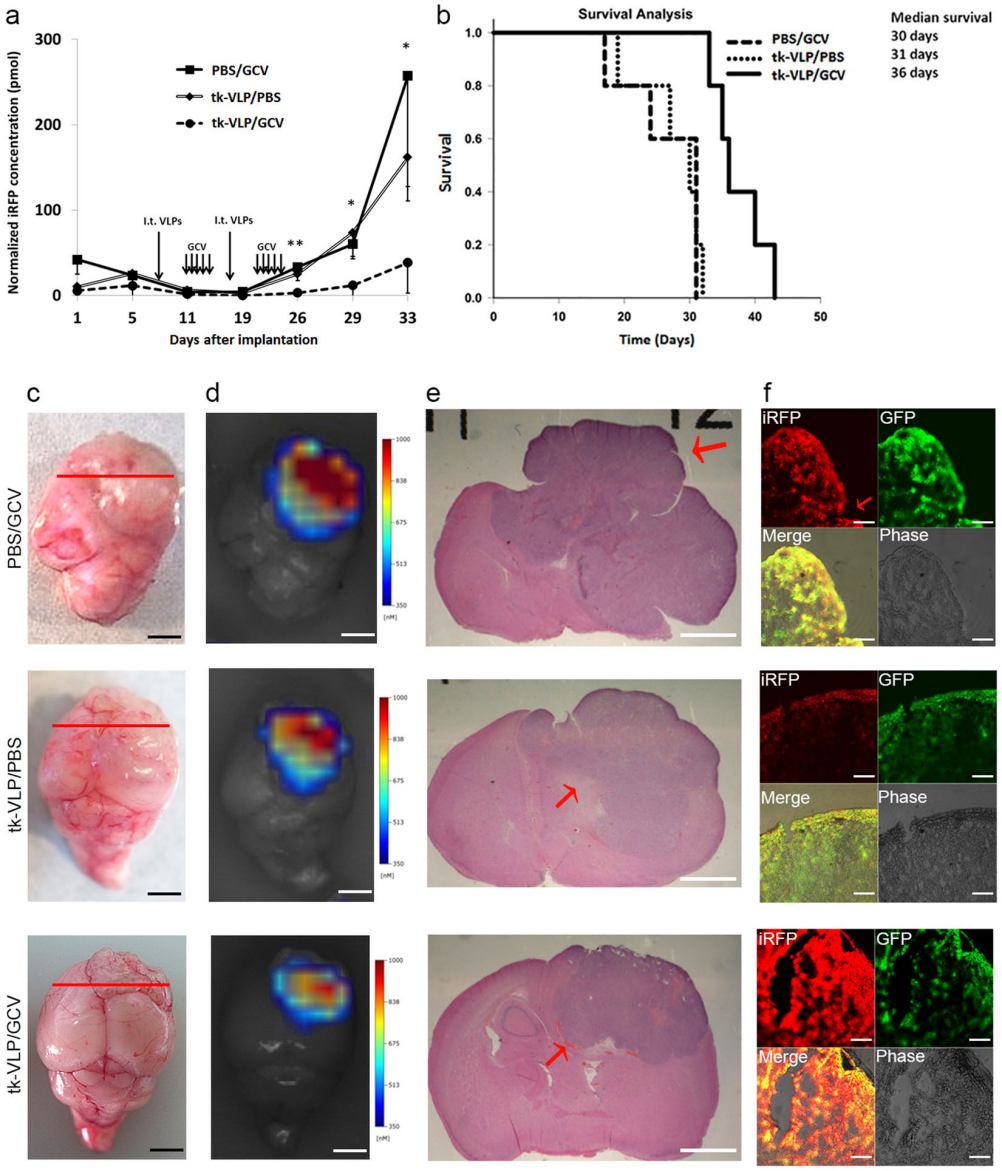
Analysis of mice with orthotopic glioma treated with tk-VLPs by intratumoral injection. (**a**) Tumor iRFP fluorescence over time. Nude mice with orthotopic glioma were injected intratumorally with PBS or tk-VLPs on day 7 and day 18 and injected intraperitoneally with GCV or PBS on various days. At different time points, the three groups of mice were subjected to *in vivo* FMT imaging. Data were means ± SD. n = 5, Kruskal–Wallis test, *p* = 0.005 ** (day 26), 0.01 * (day 29), 0.016 * (day 33). (**b**) Kaplan–Meier survival analysis of mice with orthotopic glioma treated with PBS/GCV, tk-VLP/PBS or tk-VLP/GCV combination. Log-rank test, *p* = 0.003. Analysis of tissue from a representative mouse of each group. The representative mouse of PBS/GCV, tk-VLP/PBS, and tk-VLP/GCV treatment group was euthanized at 32, 32, 34 days after tumor implantation, respectively. Shown from left to right are the gross appearance of a tumor-bearing mouse brain (**c**), scale bar = 25 mm, with a red line marking the location of brain sections taken; an *ex vivo* FMT scan of the brain (**d**), scale bar = 25 mm; an H&E-stained brain section from the area of the tumor (**e**), scale bar = 2 mm; and confocal microscopic images of double fluorescence of the tumor area (**f**), scale bar = 60 μm.

To more closely model glioma treatment in the clinical setting, we waited one week for orthotopically implanted U87-L-iRFP cells to grow in nude mice before injecting the mice with tk-VLPs intratumorally on days 7 and 18 for a total of two doses, in combination with GCV administration. Mice in this experimental treatment group (tk-VLPs/GCV) had significantly less intracranial tumor iRFP fluorescence since day 26 (19 days after treatment began and shortly after the last day of treatment) than mice in either control treatment group (PBS/GCV or tk-VLPs/PBS), as shown by *in vivo* FMT imaging (Fig. 4a and Supplementary Fig. 3a–c), and had significantly better survival (Fig. 4b). All of the control mice developed disease by day 32 of the experiment, as did all of the experimental mice by day 43. The mean weight of mice before the onset of disease did not differ between the three groups (Supplementary Table 2). Tumors were observed in the brain sections of the mice after they were euthanized upon disease onset (Fig. 4c and e), and iRFP fluorescence was detected in the tumor areas both in *ex vivo* FMT scans (Fig. 4d) and under a confocal microscope (Fig. 4f).

### Tail vein injection of tk-VLPs combined with GCV inhibits the growth of subcutaneous U87-MG tumors in mice

**Figure 5:**
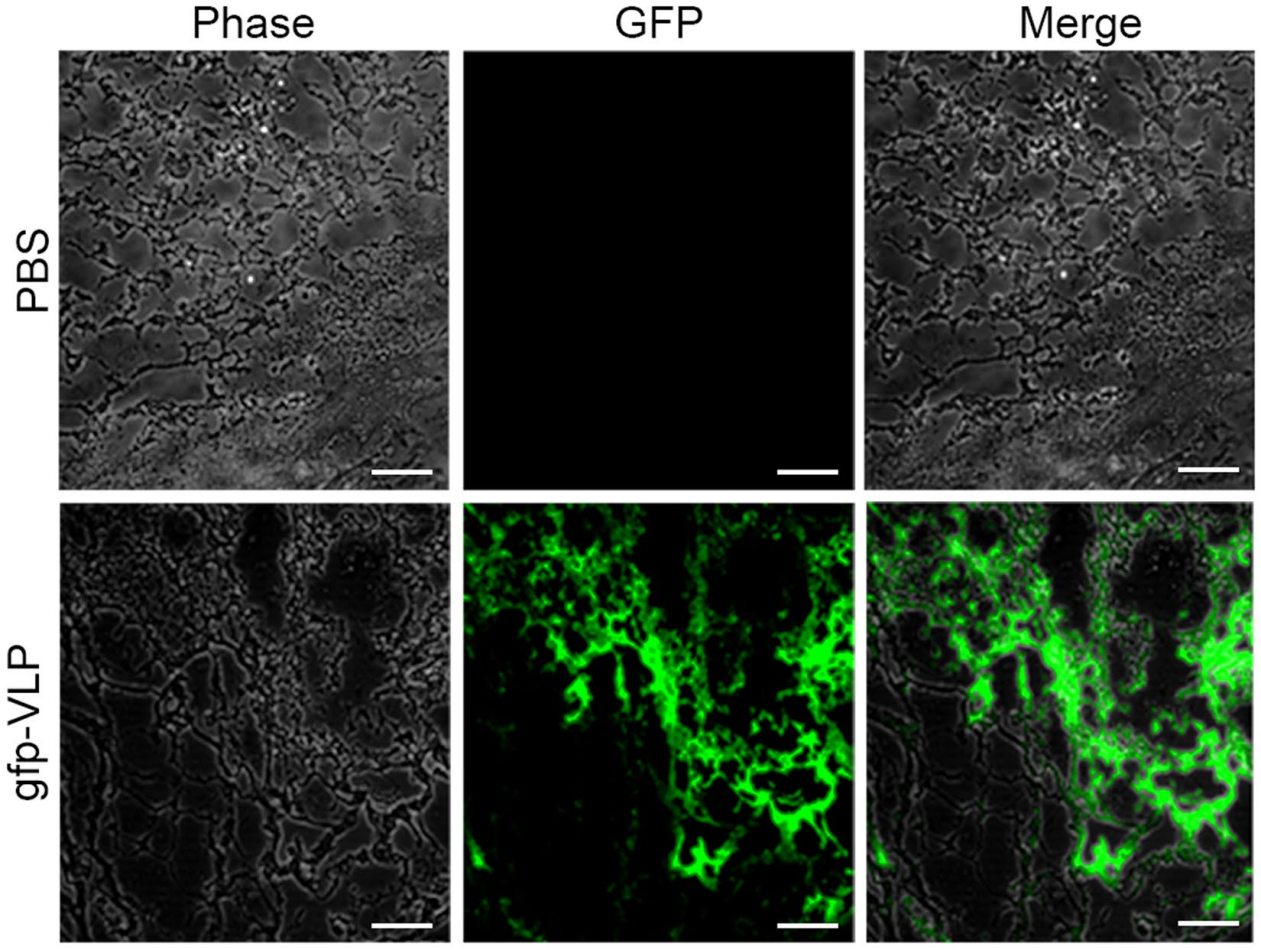
Delivery of gfp-VLPs via tail vein injection to subcutaneous glioblastoma tumor nodules in mice. PBS or gfp-VLPs were injected via tail vein into nude mice that had been inoculated subcutaneously with U87-MG cells. The resulting tumor nodule from each mouse was cryosectioned and examined by fluorescence confocal microscopy for GFP expression. Scale bar = 100 μm.

**Figure 6:**
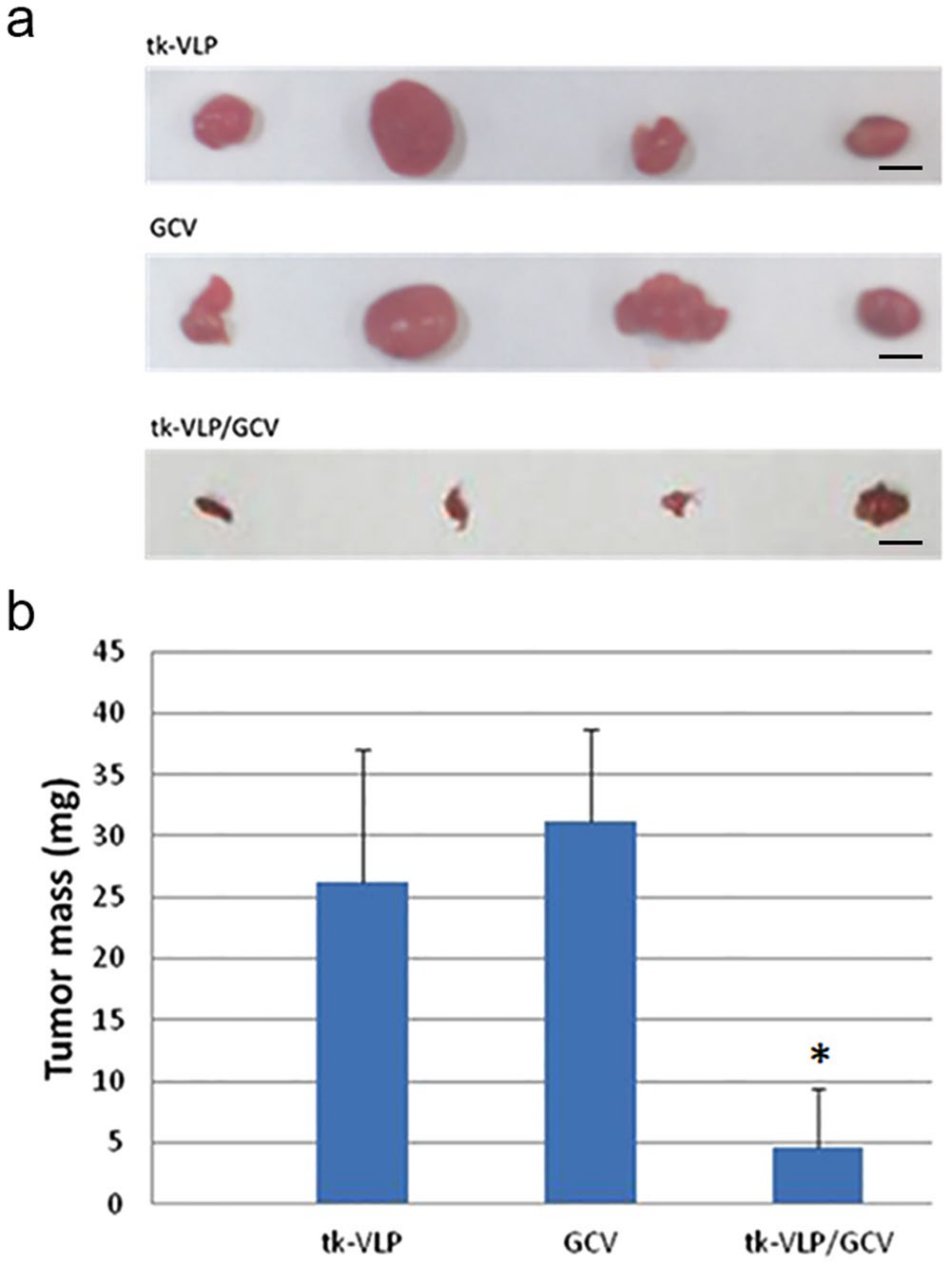
Inhibition of human glioblastoma tumor growth by tail vein–injected tk-VLPs combined with GCV treatment in a subcutaneous xenograft model. Tail vein-injected tk-VLPs, intraperitoneally injected GCV, or combined tk-VLPs/GCV were administered to nude mice bearing subcutaneous U87 human glioblastoma tumors. (**a**) Gross pictures of tumor nodules harvested on day 21 from each treatment group. Scale bar = 50 mm. (**b**) Quantification of tumor nodule weights presented as means ± SD. Combined tk-VLPs/GCV treatment exhibited a significant reduction in the weights of tumor nodules compared to tk-VLP only or GCV only treatment. n = 4, Mann-Whitney U Test, *p < 0.05.

We examined whether JCPyV VLPs being transported in the mammalian circulatory system could protect their packaged expression plasmids and reach the target U87-MG tumor cells for gene delivery and expression. Nude mice harboring subcutaneous U87 tumors were administered gfp-VLPs by tail vein injection. Subsequent histopathological examination of tissue sections from the mice revealed that only the subcutaneous U87-MG tumors expressed GFP (Fig. 5). Moreover, in nude mice receiving tk-VLPs by tail vein injection in combination with GCV treatment, the growth of subcutaneous U87 tumors was effectively inhibited (Fig. 6a and b). The mean weight of mice at the end of the experiments did not show any difference between gfp-VLP, tk-VLP, or PBS groups after tail vein injection (Supplementary Fig. 4a and b). Therefore, it may indicate that the non-TK-expressing VLPs delivered through tail vein had no effect on the mice. These results show that JCPyV VLPs protected the integrity of the packaged genes while being transported in the circulatory system of a living animal, and reached subcutaneous U87 tumors at distal sites where the genes were delivered and expressed, thereby achieving a therapeutic effect.

## Discussion

In this study, we investigated JCPyV VLPs as a potential therapeutic tool in GBM treatment by performing both *in vitro* and *in vivo* analyses. Our cell culture results show that JCPyV VLPs are able to transduce human glioblastoma cells and carry the expression plasmids for the GFP reporter and for a suicide gene into these cells for expression, achieving a cytotoxic effect with the latter plasmid type. Results from our animal experiments show that JCPyV VLPs introduced into nude mice, whether by direct intratumoral injection or through the systemic circulation, can protect the suicide gene they carry until reaching tumors formed by human glioblastoma cells and achieve inhibition of tumor growth and prolonged survival in nude mice with orthotopic glioma. In our analysis of the orthotopic mouse model of glioma, intravital FMT enabled the real-time detection of tumors formed by iRFP-expressing human glioblastoma cells and allowed the therapeutic effectiveness of suicide gene–carrying VLPs to become apparent early in the experiment. Collectively, these findings demonstrate the potential usefulness of JCPyV VLPs as a gene therapy vector for treatment of GBM.

The interaction of the JCPyV capsid with cellular receptors is crucial restriction for neurotropism of JCPyV^24^. Both the sialylated oligosaccharides as attachment receptors and serotonin 5-HT_2A_ receptors as entry receptors are necessary for productive infection by JCPyV^7,25–27^. After binding the receptors, JCPyV enters glial cells by clathrin-mediated endocytosis^28^. A crystal structural analysis of the JCPyV capsid protein VP1 in complex with a functional JCPyV recognition motif revealed extensive interactions between the external BC and HI loops of VP1 and α2,6-linked oligosaccharide lactoseries tetrasaccharide c (LSTc)^29^. A study examining the *in vivo* expression of JCPyV receptors in the human brain by immunohistochemistry detected the expression of serotonin receptors, but not LSTc, in oligodendrocytes and astrocytes^30^. Other studies found that besides α2,6-linked sialic acids, JCPyV also uses α2,3-linked sialic acids on N-linked glycoproteins to infect glial cells^26,31^. Recently, it has been reported that amino acid sequences of JCPyV VP1 isolated from PML patients may have adaptive mutations. The VP1-derived VLPs carrying these mutations might favor brain or CNS cells invasion in a sialic acid binding-independent manner^32–35^. Malignant gliomas lack cell surface α2,6-linked sialic acids but significantly overexpress α2,3-sialyltransferase and α2,3-linked cell surface sialic acids in contrast to normal human glial cells^36,37^. In addition, relative to human normal fetal astrocytes, glioma cells overexpress seven 5-HT receptors, including the 5-HT_2_ receptor family which mediates JCPyV entry^38^. Taken together, the above evidence indicates that JCPyV VLPs may enter human glioblastoma cells through the use of JCPyV attachment and entry receptors specifically over-expressed in glioma cells. Our *in vitro* and *in vivo* studies also demonstrate that JCPyV VLPs are indeed able to transduce human glioblastoma U87 cells and deliver the GFP reporter gene into tumor cells for expression.

This is the first study to test JCPyV VLPs in animals for the purpose of gene therapy for human malignant glioma. In the orthotopic xenograft tumor model, we tested two approaches for administration of JCPyV VLPs: mixing tk-VLPs with U87-L-iRFP cells and injecting the mixture into the right brain of nude mice, or two stereotactic intratumoral injections of tk-VLPs one week after tumor cell implantation. With respect to the first approach, delivery of control VLPs or tk-VLPs as an intracranially implanted mixture with tumor cells in combination with GCV treatment, one tk-VLP–implanted mouse survived to day 70 in good health and without detectable tumor iRFP fluorescence in consecutive FMT scans, and the median survival for this group was longer than that of mice treated by intratumoral injections. This difference could be due to the higher gene transduction efficiency of tk-VLPs being mixed with and thus being in close contact with the tumor cells, or the superior tumor-inhibitory effect of giving the tk-VLPs/GCV treatment early, before the implanted tumor had a chance to grow. The mean weight of mice before the onset of disease did not differ between the treatment and control groups. This may be because the suicide gene treatment was administered locally to the intracranial tumor site and consequently was able to avoid off-target toxicity; alternatively, JCPyV VLPs may be unable to transduce mouse cells. With respect to our second approach, that of administering tk-VLPs as intracranial intratumoral injection, it was also effective in bringing about inhibition of tumor growth and prolonged survival. Our endpoint anatomical and histopathological examination of the brains showed that all of the tumors were centered on the needle insertion point in the right brain, supporting the colocalization of the two stereotactic intratumoral VLP injections with the tumors. What we observed in our cell–tk-VLP mixture and intratumoral tk-VLP injection experiments strongly suggests that gene therapy is more effective when administered as soon as possible. HSV-TK/GCV suicide gene therapy has been reported to enhance the sensitivity of glioblastoma cells to chemotherapy and radiation therapy for a synergistic effect^39–41^, further supporting the idea that such combination therapy may be considered earlier during treatment.

The endpoint of our animal experiments was based on the appearance of neurological symptoms, allowing the potential early benefits of a gene therapy approach to be assessed. Although whether tumor progression or neurological symptoms had occurred was interpreted by the experimenter who was blinded to treatment allocation, interpretation bias might not have been entirely avoidable. Therefore, *in vivo* time-series FMT imaging to measure the iRFP fluorescence of nude mice with orthotopic glioma was conducted in order to objectively assess the effect of tk-VLP/GCV gene therapy on the tumors. To test the reliability of *in vivo* FMT tumor imaging, we performed *ex vivo* FMT imaging followed by histopathological analysis. The results showed that the location of the FMT fluorescence signals was consistent with the brain location of the tumors as seen with the naked eye and with a microscope, and sections of the tumors also exhibited double fluorescence under a confocal microscope, indicating that the fluorescence signals detected in FMT originated from the tumors. In the intratumoral tk-VLP injection experiment, *in vivo* FMT revealed a significant difference in quantified tumor fluorescence between the experimental and control groups from day 26 on, the next day after completing the treatment, with the tumor fluorescence of the control groups continuing to increase rapidly. Unlike survival analysis, in which therapeutic efficacy can be determined only at the end of the experiment, *in vivo* time-series FMT monitoring allows a difference in the effectiveness of treatment to be discerned midway through the experiment so that the treatment strategy can be adjusted accordingly. For instance, when a gradual rise in iRFP fluorescence was observed in intratumoral tk-VLP–injected mice on days 26 through 33, possible responses included extending the GCV treatment period or adding a third intratumoral injection of tk-VLPs along with further GCV treatment, and the results of such adjustments could be tracked by intravital FMT. In the present study, we used FMT to detect and quantify the fluorescence of the iRFP marker protein that was stably expressed in tumors in living mice, and this system enables the prediction of tumor response to gene therapy during an experiment. For research on new drugs or new gene therapy approaches for orthotopic glioma in small animals, the combination of FMT and the iRFP tumor marker permits rapid, time-series imaging analysis of tumor response, and can be used to facilitate high-throughput drug screening and the development and optimization of new gene therapies.

GBM is rarely associated with extracranial metastases, and in the majority of recurrent cases, tumors form at the original tumor location. Consequently, research on gene therapy for GBM has focused on local therapy strategies such as intratumoral injection, including several phase I to phase III clinical trials of malignant glioma gene therapy^42–45^. However rare, extraneural metastases occur in approximately 0.4% to 2% of GBM patients^46^, and this small group of patients have a dismal prognosis and no effective treatment options. Also, among organ transplants involving 69 organ donors with GBM, the incidence of donor-transmitted GBM in the transplant recipients was found to be 1.4%^47^. Therefore, the most likely mechanism of extraneural metastasis of GBM is blood vessel invasion and hematogenous spread. In one study, blood cell samples from GBM patients were screened by immunostaining for glial fibrillary acidic protein (GFAP) as a GBM marker, and GFAP-positive cells harboring glioblastoma-associated genomic aberrations were present in 29 out of 141 (21%) GBM patients, not in healthy volunteers^48^. This finding suggests that hematogenous dissemination of tumor cells is an intrinsic feature of GBM and occurs much more frequently in patients than overt extraneural metastases. Our study showed that after injection via the tail vein, JCPyV VLPs were able to protect the packaged suicide gene while being transported in the blood circulation, target distal subcutaneous glioblastoma tumors, and, in combination with GCV, effectively inhibit tumor growth. Future research can test using JCPyV VLPs to package a GFAP promoter–driven therapeutic gene to target circulating tumor cells or overt extraneural metastases in patients with GBM.

In conclusion, the current study demonstrated that local administration of tk-VLPs in the tumor area, supplemented by systemic GCV treatment, can effectively inhibit the growth of orthotopic tumors from human malignant glioblastoma cells in nude mice and prolong the survival of the mice. We also showed the usefulness of *in vivo* time-series FMT monitoring and quantification of tumor cells stably expressing the iRFP fluorescent marker, which allows experimenters not only to assess the effectiveness of gene therapy in an orthotopic glioma living animal model but also to predict tumor response to gene therapy earlier than and in accordance with survival analysis. Furthermore, tail vein–injected tk-VLPs can target human glioblastoma cells in nude mice and, when combined with GCV, inhibit the growth of subcutaneous tumors formed by those cells. These results demonstrate the potential of neurotropic JCPyV VLPs to serve as a gene therapy vector for treatment-refractory GBM.

## Materials and Methods

### Cell lines

U87-MG cells (ATCC, HTB-14) were infected with lentiviral vectors (from the National RNAi Core Facility, Taiwan) bearing the coding sequences of iRFP (piRFP; Addgene, USA) and GFP (pLKO_AS3w.tGFP; RNAi Core Lab, Taiwan) and then selected for double-fluorescent cells using a flow cytometer (FACSAria III; BD Biosciences). Selected cells were further cultured to yield a stable cell line, U87-L-iRFP. U87-MG and U87-L-iRFP cells were cultured in DMEM (Gibco) supplemented with 10% fetal bovine serum (Gibco) and 1% penicillin–streptomycin–amphotericin B (Gibco) at 37 °C in 5% CO_2_. The double fluorescence of U87-L-iRFP cells was observed by using a confocal microscope (LM-510; Zeiss, Germany). Photos were taken using Zeiss LSM 510 laser confocal microscope (Carl Zeiss, Thornwood, NY) with excitation of GFP at 488 nm laser and excitation of iRFP at 633 nm laser. Plan-Neofluar 20X/0.5Ph2 objective was used and magnification was 200 ×. LSM 510 basic software was used for image capture and analysis.

### Preparation of VLPs

The JCPyV VP1 expression plasmid ∆pFlag-JC VP1^9^ was co-transformed with either of pEGFP-N3 (Clontech, Palo Alto, California, USA) or pUMVC1-tk (Aldevron, Fargo, North Dakota, USA) into JM109 E. coli (Promega, Madison, Wisconsin, USA). VLPs carrying plasmid DNA for expressing GFP and HSV-TK, referred to as gfp-VLPs and tk-VLPs, respectively, were prepared as previously described by Chen *et al*.^10^.

### Transduction of U87-MG cells with gfp-VLPs

U87-MG cells were first washed twice with PBS and resuspended in 50 μl of PBS. Next, 10 μg of control VLPs or gfp-VLPs were added to the cells, and mixture was incubated at 4 °C for 1 hour with gentle shaking every 10 min. Afterwards, the cells were washed twice with cool PBS to remove free VLPs, placed in complete medium, and then incubated at 37 °C in 5% CO_2_ for 72 hours. The expression of GFP in the transduced cells was then visualized with a fluorescence microscope (Carl Zeiss, Thornwood, NY).

### Cytotoxicity assays

U87-MG cells were seeded in a 96-well flat-bottom plate (TPP Techno Plastic Products AG, Trasadingen, Switzerland) at 4 × 10^3^ cells per 100 μl of complete medium per well and cultured overnight. The culture medium was removed and then either 1 μg of control VLPs or tk-VLPs, or 50 μl of PBS was added to each well and allowed to incubate with the cells at room temperature for 1 h with gentle shaking of the plate every 15 min. Afterward, the culture medium was added till 100 μl per well and the cells were incubated at 37 °C in 5% CO_2_ overnight. Then culture medium was removed and 100 μl of GCV (InvivoGen, San Diego, California, USA) or PBS was added to each well and the cells were incubated in 5% CO_2_ at 37 °C with or without 50 μg/ml of GCV. At 72 hours post-transduction, the culture medium was changed to 100 μl per well of fresh complete medium containing 10 μl of CCK-8 solution (Sigma-Aldrich, Saint Louis, Missouri, USA), and the cells were incubated at 37 °C in 5% CO_2_ for 1 hour. Absorbance at 450 nm was then measured using a microplate reader (Thermo Fisher Scientific, Waltham, MA, USA), and cell viability was calculated.

### Orthotopic murine models of human glioblastoma

All animal experiments were approved by the Institutional Animal Care and Use Committee of National Chung Cheng University (IACUC Approval No. 1040701) and were conducted in accordance with relevant regulations. Mice that exhibited rapid weight loss (10% or more in three days) or onset of significant neurological symptoms such as seizures, impaired balance, hemiplegia, and bleeding from the eyes were considered to have reached the experimental endpoint and were euthanized by CO_2_ asphyxiation.

#### Mice injected with U87 cells pre-mixed with tk-VLPs

Before surgery, six- to eight-week-old female nu/nu mice purchased from BioLASCO Taiwan Co., Ltd. (Taipei, Taiwan) were given atropine (Tai Yu Chemical & Pharmaceutical Co., Taiwan) and then zoletil (Virbac, France) by intraperitoneal injection at 0.12 mg and 5 mg per 100 g of body weight (BW), respectively. With the use of gas anesthesia masks, the mice were then administered isoflurane, 4% induction 1–2% maintenance, to achieve stable anesthesia. For each anesthetized mouse, the head was secured in a stereotactic head frame (Harvard Apparatus, Holliston, Massachusetts, USA), the surgical site was sterilized with Betadine, and an incision was made with a scalpel along the midline of the scalp to expose the skull, and the skull was gently swabbed with a cotton swab, allowing the bregma to be located. With the bregma as a reference point, a bone drill was used to make a small hole through the skull at a location 2.0 mm to the right along the coronal suture and 1 mm anterior. Five million U87-L-iRFP cells were mixed with 10 μg of either control VLPs or tk-VLPs for a total volume of 5–7 μl in Hank’s balanced salt solution (Thermo Scientific) and kept on ice until use. The cell–VLP mixture was slowly injected through the hole into the brain at a depth of 2.5 mm via a syringe fitted with a 25-gauge needle (Hamilton Co., USA). When the injection was completed, the needle was left in place for 5 min for blood clotting to occur before the needle was slowly withdrawn, and the incision was then closed with sutures. The day of tumor cell injection was defined as day 0. Beginning on day 3, GCV was given daily by intraperitoneal injection at 50 μg/g BW for five consecutive days and a total of five doses.

#### Mice with orthotopic glioma injected intratumorally with tk-VLPs

After an orthotopic glioma model was established in nude mice through implantation of 1 × 10^6^ U87-L-iRFP cells at a depth of 3 mm, the mice were injected intratumorally with PBS or 10 μg of tk-VLPs in 5 μl by the same method at the same location on days 7 and 18. Beginning on day 10 and again on day 21, the mice were injected intraperitoneally with PBS or with 50 μg/g BW of GCV daily for five consecutive days and a total of ten doses.

#### FMT imaging

*In vivo* FMT (FMT 4000; PerkinElmer, USA) was conducted once or twice per week, in which mice were given an isoflurane/oxygen gas mixture and anesthesia was maintained throughout. Each anesthetized mouse was weighed and placed in an imaging cassette in the prone position and taken into the FMT imaging chamber through a docking system, regulated at 37 °C. After the built-in LED light source was used to generate a 2D planar fluorescence reflectance image, the scan field was set to the head region, and 3D scanning was initiated with laser excitation at 680 nm for the detection of iRFP. Scans were conducted at 80 mW excitation power with 2–5 s exposure time, for 5–6 min per mouse, using excitation filters of 680 nm with 6–22 nm bandwidths and emission filters of 690–740 nm. TrueQuant v3.0 software (PerkinElmer) was used to reconstruct collected fluorescence data, and regions of interest in the brain were circled for the quantification of fluorescence signal. The fluorescence signals of brain regions of interest were compared with those of known concentrations of the fluorescent imaging agent AngioSense 680 (PerkinElmer), and the resulting fluorescence quantification data were expressed as corrected picomolar amounts of iRFP.

#### Analysis of tissue sections

After euthanasia by CO_2_ asphyxiation at the experimental endpoint, tumor-bearing brains were removed from euthanized mice for *ex vivo* FMT imaging, and then sectioned coronally at the midline of tumor regions. Half of sections from each block were fixed in 10% formalin and the other half embedded in optimal cutting temperature (OCT) compound. Formalin-treated specimens were embedded in paraffin, sectioned, and stained with hematoxylin and eosin (H&E). Specimens treated with OCT were directly cut with a cryotome into coronal sections 7–12 μm in thickness, which were then stored at −20 °C until they were examined for GFP and iRFP fluorescence with a confocal microscope (Zeiss LM-510).

### Subcutaneous xenograft models of human glioblastoma

#### Tail vein injections of gfp-VLPs

Five million U87-MG cells were injected subcutaneously in the right flank of nude mice. Seven days later, the mice were injected in the tail vein with 70 μg of gfp-VLPs or with PBS every other day for a total of six injections. On day 20, mice were weighed and tumor nodules were removed, cryosectioned, and examined for GFP expression under a confocal microscope.

#### Systemic administration of tk-VLPs

Nude mice were injected subcutaneously in the right flank with 5 × 10^6^ U87-MG cells. Beginning on the same day, the mice were injected in the tail vein with 70 μg of tk-VLPs every other day, and beginning next day, injected intraperitoneally with 60 mg/kg BW of GCV every other day, for ten total injections each. Control treatments consisted of tk-VLP injections only or GCV injections only. On day 21, mice were weighed and subcutaneous tumor nodules were surgically removed and photographed, and their weights were measured.

### Statistical analysis

For fluorescence signals quantified by FMT imaging in picomoles, body weights of mice, and tumor weights, differences in median values were examined by the Mann–Whitney U test for two independent samples or by the nonparametric Kruskal–Wallis test for three independent samples. For survival curves, Kaplan–Meier survival analysis was performed along with the log-rank test for differences. A *P* value < 0.05 indicated a statistically significant difference.

### Data availability

All data generated and analysed during this study are included in this published article and its Supplementary Information files.

## Electronic supplementary material

Supplementary Information
