# Supplementary material for: Gene therapy for human glioblastoma using neurotropic JC virus-like particles as a gene delivery vector
Source: Sci Rep. 2018 Feb 2;8:2213. doi: 10.1038/s41598-018-19825-w (PMC5797127; doi:10.1038/s41598-018-19825-w)
Supplement: Supplementary file 1 — Supplementary Information [file 41598_2018_19825_MOESM1_ESM.pdf]

Gene therapy for human glioblastoma using neurotropic JC virus-like particles as a gene delivery vector

Chun-Nun Chao[1,2], Yu-Hsuan Yang[1,2], Mu-Sheng Wu[2], Ming-Chieh Chou[1,2], Chiung-Yao Fang[3], Mien-Chun Lin[2,4], Chien-Kuo Tai[2], Cheng-Huang Shen[4], Pei-Lain Chen[5], Deching Chang[2,*] & Meilin Wang[6,*]

[1]Department of Pediatrics, Ditmanson Medical Foundation Chiayi Christian Hospital, Chiayi, Taiwan.

[2]Institute of Molecular Biology, National Chung Cheng University, Chiayi, Taiwan.

[3]Department of Medical Research, Ditmanson Medical Foundation Chiayi Christian Hospital, Chiayi, Taiwan.

[4]Department of Urology, Ditmanson Medical Foundation Chiayi Christian Hospital, Chiayi, Taiwan.

[5]Department of Medical Laboratory Science and Biotechnology, Central Taiwan University of Science and Technology, Taichung, Taiwan.

[6]Department of Microbiology and Immunology, School of Medicine, Chung-Shan Medical University and Clinical Laboratory, Chung-Shan Medical University Hospital, Taichung, Taiwan.

Supplementary Fig 1. Stable expression of GFP and iRFP in U87-L-iRFP cells. Confocal microscopic images are shown for the GFP and iRFP fluorescence of U87-MG and U87-L-iRFP cells. Scale bar = 60 μm.

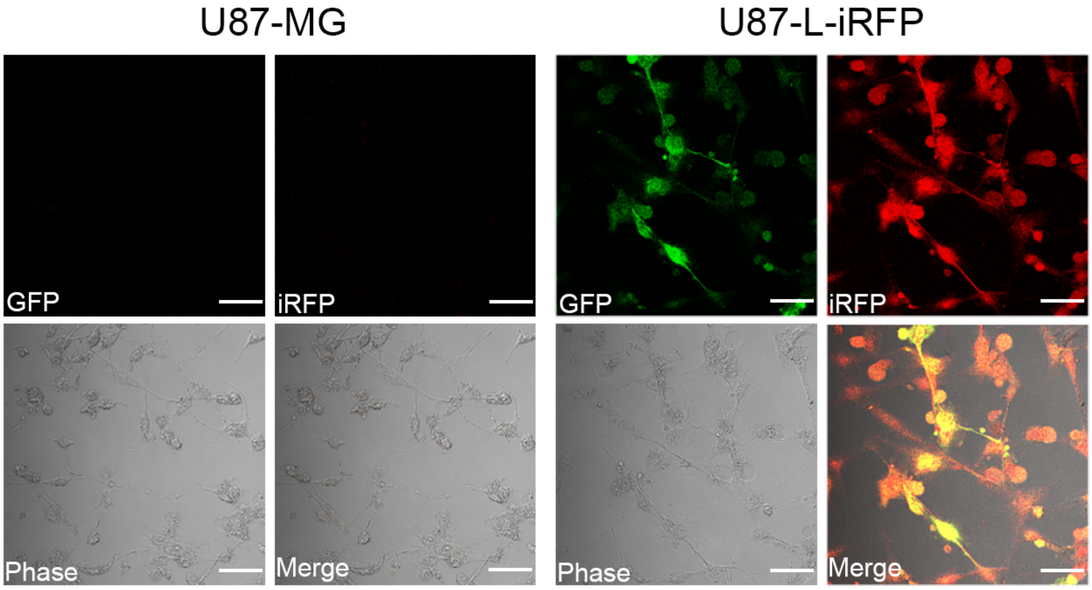

Supplementary Fig 2. *In vivo* FMT images of mice injected with U87 glioblastoma cells pre-mixed with tk-VLPs in an orthotopic glioma model. Mice were intracranially implanted with U87-L-iRFP cells mixed with either control VLPs (U87-L-iRFP + VLP) or tk-VLPs (U87-L-iRFP + tkVLP) and monitored between days 36 and 49 through FMT.

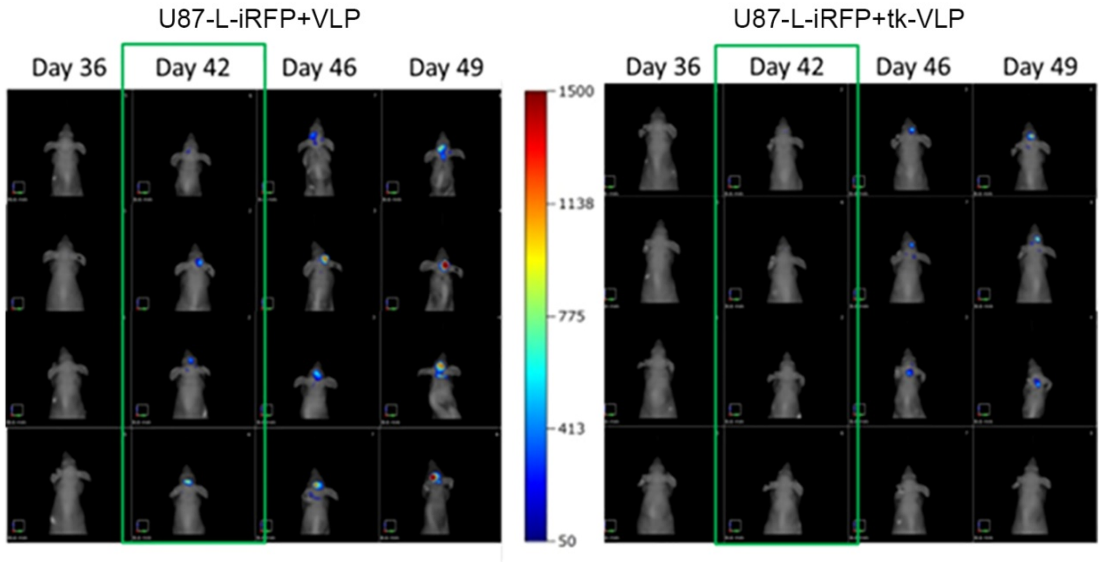

Supplementary Fig 3. *In vivo* FMT images of mice with orthotopic glioma treated by intratumoral injection of tk-VLPs. (**a**) Mice treated with intratumoral PBS and intraperitoneal GCV (PBS/GCV) imaged at different time points. (**b**) Mice treated with intratumoral tk-VLPs and intraperitoneal PBS (tkVLP/PBS) imaged at different time points. (**c**) Mice treated with intratumoral tk-VLPs and intraperitoneal GCV (tkVLP/GCV) imaged at different time points.

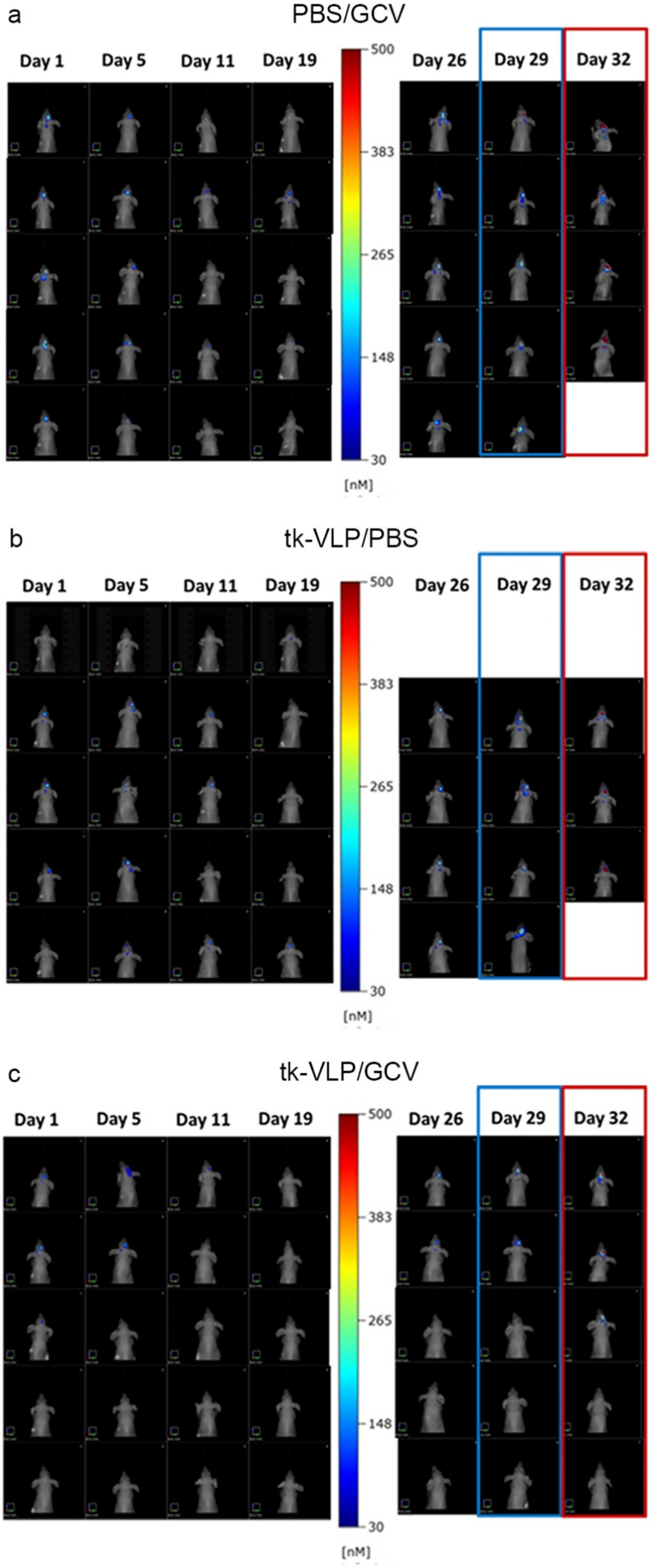
a
PBS/GCV
Day 1
Day 5
Day 11
Day 19
Day 26
Day 29
Day 32
500
383
265
148
30
[nM]
b
tk-VLP/PBS
Day 1
Day 5
Day 11
Day 19
Day 26
Day 29
Day 32
500
383
265
148
30
[nM]
c
tk-VLP/GCV
Day 1
Day 5
Day 11
Day 19
Day 26
Day 29
Day 32
500
383
265
148
30
[nM]

Supplementary Fig 4. The mean weight of mice harboring subcutaneous U87-MG tumors in tail vein injection groups at the end of the experiment. (a) PBS or gfp-VLPs were injected via tail vein into nude mice that had been inoculated subcutaneously with U87-MG cells. Body weights of mice from each group were quantified on day 20 and presented as means ± SD in a bar graph. n = 3, Mann-Whitney U Test, p = 0.127 (b) Tail vein-injected tk-VLPs, intraperitoneally injected GCV, or combined tk-VLPs/GCV were administered to nude mice bearing subcutaneous U87-MG tumors. Body weights of mice from each group were quantified on day 21 and presented as means ± SD, n = 4, in a bar graph. Kruskal–Wallis test showed no difference, p = 0.4, when comparing weights between PBS group, GCV group, and gfp-VLP and tk-VLP injected groups.

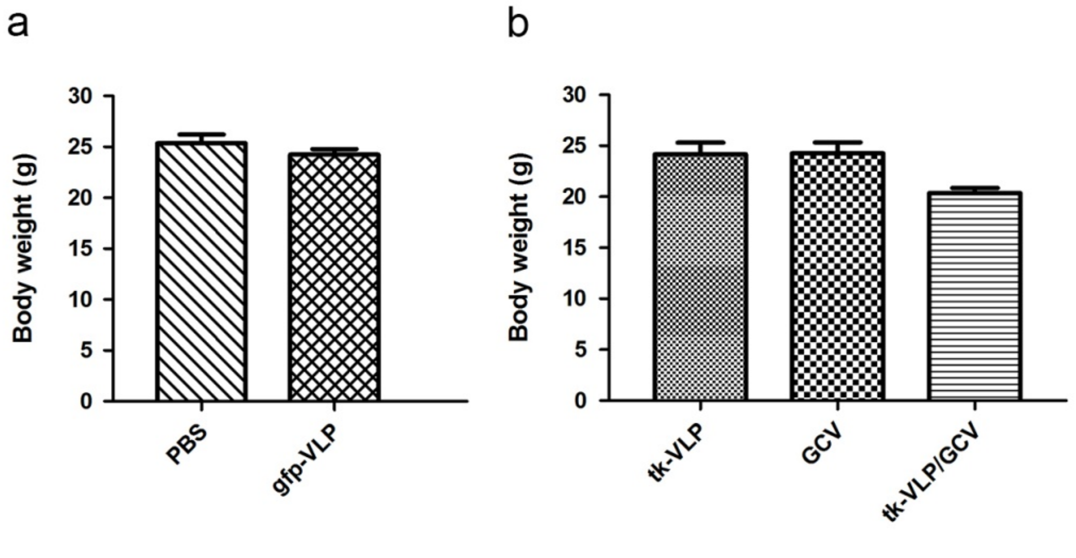

Supplementary Table 1. Body weight (grams) of mice in pre-mixed groups since implantation of cell-VLP mixtures.

Day	VLP	tk-VLP	p-value
1	19.55±1.06	19.60±0.42	0.561
8	21.20±1.51	20.48±1.06	0.564
14	23.53±1.55	22.73±1.24	0.149
22	23.68±1.59	22.25±1.03	0.149
28	25.25±2.33	25.38±1.16	0.663
36	25.43±3.52	25.10±1.32	0.564
42	23.18±3.81	24.53±2.09	0.386
46	19.03±2.07	24.08±3.45	0.146
49	15.18±1.47	23.00±5.02	0.043

Data are shown as means ± SD. n = all live animals at each time point <= 4.

Differences were assessed by Mann-Whitney U test.

Supplementary Table 2. Body weight (grams) of mice in intratumoral injection groups after tumor cell implantation.

Day	PBS/GCV	tk-VLP/PBS	tk-VLP	p-value
1	22.88±1.55	22.94±2.11	24.16±1.91	0.399
5	21.54±2.11	23.74±2.61	24.14±1.75	0.125
11	22.34±3.32	22.96±3.70	25.16±1.86	0.281
19	20.78±3.89	23.28±3.97	25.28±2.20	0.164
26	17.45±1.31	21.50±2.75	25.76±2.39	0.010

Data are shown as means ± SD. n = all live animals at each time point <= 5.

Differences were assessed by Kruskal–Wallis test.
